# Smartphone Apps About Crystal Methamphetamine (“Ice”): Systematic Search in App Stores and Assessment of Composition and Quality

**DOI:** 10.2196/10442

**Published:** 2018-11-21

**Authors:** Cath Chapman, Katrina Elizabeth Champion, Louise Birrell, Hannah Deen, Mary-Ellen Brierley, Lexine A Stapinski, Frances Kay-Lambkin, Nicola C Newton, Maree Teesson

**Affiliations:** 1 National Health and Medical Research Council Centre of Research Excellence in Mental Health and Substance Use National Drug and Alcohol Research Centre University of New South Wales Sydney Australia; 2 Department of Preventive Medicine Feinberg School of Medicine Northwestern University Chicago, IL United States; 3 Priority Research Centre for Brain and Mental Health The University of Newcastle Newcastle Australia

**Keywords:** internet, methamphetamine, mobile phone, review, substance-related disorder

## Abstract

**Background:**

Amid considerable community concern about the prevalence and harms associated with the use of crystal methamphetamine (“ice”), the increased use of smartphones to access health information and a growing number of available smartphone apps related to crystal methamphetamine, no previous reviews have examined the content and quality of these apps.

**Objective:**

This study aims to systematically review existing apps in the iTunes and Google Play Stores to determine the existence, composition, and quality of educational smartphone apps about methamphetamines, including ice.

**Methods:**

The iTunes and Google Play Stores were systematically searched in April 2017 for iOS Apple and Android apps, respectively. English-language apps that provided educational content or information about methamphetamine were eligible for inclusion. Eligible apps were downloaded and independently evaluated for quality by 2 reviewers using the Mobile Application Rating Scale (MARS).

**Results:**

A total of 2205 apps were initially identified, of which 18 were eligible and rated using the MARS. The mean MARS quality total score for all rated apps was 3.0 (SD 0.6), indicating poor to acceptable quality. Overall, mean scores were the highest for functionality (mean 4.0, SD 0.5) and lowest for engagement (mean 2.3, SD 0.7).

**Conclusions:**

This study demonstrates a shortage of high-quality educational and engaging smartphone apps specifically related to methamphetamine. The findings from this review highlight a need for further development of engaging and evidence-based apps that provide educational information about crystal methamphetamine.

## Introduction

In recent years, there has been a marked concern about the use of methamphetamine, particularly crystal methamphetamine (or “ice”), and the considerable harms associated with its use for both individuals, their loved ones, and communities. According to the latest National Drug Strategy Household Survey [1], approximately 1.4% of the Australian population (aged ≥14 years) reported past year use of methamphetamine (including ice) in 2016. Although these data indicate that rates of methamphetamine use in the general population have remained fairly stable over the last decade, data from several sources suggest that there have been considerable changes in the patterns of use and harmful use; these include increases in the number of regular users who report using crystal methamphetamine (ice), as opposed to powder (speed) as their main form of methamphetamine [1,2], regular and dependent users [3], and harms associated with use [4,5]. Data from several sources suggest that rates of crystal methamphetamine use in regional and rural areas of Australia are of particular concern [1,6,7], and in 2016, 40% of Australians rated methamphetamine (including ice) as the drug of most concern, compared with 16% in 2013 [1]. In addition, international data indicate increasing use and harms associated with methamphetamines. Methamphetamines now account for 11% of overdose deaths in the United States [8], and market analyses in several parts of the globe indicate an increase in the use of methamphetamines (including ice) in recent years [9].

A key component of addressing community concern around illicit drugs, including ice, and preventing use and related harms, is the provision of accurate and evidence-based information, resources, and support. The use of the internet, smartphone apps, and mobile technology is a key means of disseminating public health information to the community and facilitating broad reach and engagement. Smartphone devices are now widely used, with 64% of the United States and 74% of the Australian population owning a device in 2015-2016 [10,11], and 62% of smartphone owners reporting that they used their phone to access health-related information in the past year [11]. Like internet-based and Web-based interventions, smartphone apps offer numerous advantages in terms of addressing public health issues [12,13] such as increased accessibility, portability of information, low costs, anonymity, and the ability to provide tailored feedback and support.

Over the past decade, there has been a dramatic increase in the number of smartphone apps designed to address health-related issues [14], with >165,000 health apps available for download in 2015 [15]. Systematic reviews of smartphone apps have been conducted in a range of health domains, including depression [16], anxiety [17], bipolar disorder [18], smoking cessation [19,20], nutrition [21], diabetes management [22], suicide prevention [23], health information seeking for cancer [24], and psychology or general mental health [25-27]. The majority of these reviews focused on the quality and content of apps and concluded that the existing apps vary in quality, with few grounded in scientific evidence. Nonetheless, the results from these reviews provide useful information about the features and functionality of high-quality apps and an increased understanding of community information and support needs. Thus, they serve to guide the future development of apps, as well as future research.

When looking at the substance use field, systematic reviews and content analyses of available apps that specifically target illicit drug use are sparse. We identified several reviews of apps that target alcohol use and related behaviors [28-30], or addiction and addictive behaviors in general [31]. We found one review that specifically reviewed apps that *promote* illicit drug use [32], and one review that analyzed illicit drug overdose apps [33]. In addition, we identified 2 systematic reviews of Web-based and mobile interventions targeting problematic substance use [12,13], both of which reviewed published intervention studies rather than available apps *per se*. However, we were unable to identify an existing systematic review of publicly available apps targeting either illicit drug use in general or crystal methamphetamine specifically. This is despite the fact that a marked number of apps related to crystal methamphetamine exist, amid increasing community concern about ice use and related harms. Therefore, this study aims to systematically review existing apps in the iTunes and Google Play Stores to determine the existence, composition, and quality of educational smartphone apps about methamphetamines, including ice. We anticipate that the results from this review will inform future development and research in this area.

## Methods

### Search Strategy

Adopting similar methodology to that used in previous reviews of smartphone apps [20,23], the Australian Google Play Store for Android phone apps and Australian iOS iTunes Store for Apple iPhone apps were searched in April 2017. A comprehensive list of keywords, including common street names for crystal methamphetamine, were used including “crystal methamphetamine,” “methamphetamine,” “crystal meth,” “ice drug,” “meth,” “shabu,” “tina,” “glass,” “illegal drugs,” and “illicit drugs.” Given the changes in app availability that occurs day-to-day, all searches were undertaken on the same day.

### Eligibility Criteria and App Selection

Free and paid apps containing educational or information-based content related to methamphetamines, including ice, were included if they could be downloaded through the official Android or iOS store. Apps did not have to exclusively focus on crystal methamphetamine to be eligible for inclusion, rather they had to include some relevant educational content about ice. Apps were excluded if they were in a language other than English, if their content did not relate to crystal methamphetamine at all, if they did not include educational content about methamphetamine (eg, gaming apps and apps promoting drug use), or they were an audiobook (ie, voice recordings). After removing duplicate apps, initial screening of the titles and descriptions of identified apps was conducted by one author (KEC) using the eligibility criteria mentioned above to identify potentially relevant apps. Potentially eligible apps were then downloaded onto their respective devices (iPhone or Android) and independently assessed by 2 reviewers (LB and HD) to confirm eligibility. Eligibility for inclusion in the review was assessed by both raters in relation to predetermined criteria. Apps were excluded if they were no longer available for download; did not include educational content; were not accessible once downloaded; or were not able to be downloaded. Any discrepancies in eligibility screening between the 2 raters were resolved by consensus.

### App Classification and Quality Rating Tool

The quality of the apps was evaluated using the Mobile Application Rating Scale (MARS) [34]. Increasingly used in the mobile health field [20,35], the MARS is designed to collect descriptive and technical information about the app (App Classification) and assess the quality of the app (App Quality Ratings). The app classification information includes name, brief description, version, developer, costs, platform, focus of the app, theoretical background, affiliations (commercial, government, nongovernment organization, or university), target age group, and technical aspects (eg, ability to send reminders or share on social media). A 23-item Quality Rating scale assesses the app quality across 5 dimensions—engagement (interactivity, interest, and suitability for target audience; 5-items), functionality (ease of use and navigation; 4-items), esthetics (layout, graphics, and visual appeal; 3-items), information quality (accuracy, evidence base, and credibility; 7-items), and subjective quality (likelihood to use and recommend; 4-items). Responses are made on a 5-point scale (1=“inadequate” to 5=“excellent”), with mean scores calculated for each dimension. An overall mean quality rating score is calculated by combining the mean scores for the first 4 subscales, excluding the subjective quality. In addition, the mean of the subjective quality items is calculated to produce a separate subjective quality total score. The subjective ratings provide a measure of the raters’ perceptions of the apps guided by objective anchors and focused on the relevance for people who might benefit from the app (rather than on themselves).

A total of 18 eligible apps were rated independently by 2 assessors (LB and HD) according to the MARS. Both assessors watched a Web-based training video provided by the scale’s developers. The training video comprehensively explained the purpose of each of the subscales, as well as demonstrating how to rate items (with reference to real-world examples), and scoring instructions. Prior to rating the apps, assessors engaged with each app for, at least, 10 minutes, as recommended by the scale developers [34]. The information subscale relies on the raters’ expertise and the availability of published studies to judge the accuracy and evidence base of the provided information. Hence, both raters held, at least, an honors degree in psychology and had relevant experience in drug and alcohol research, allowing them to assess whether the information provided was relevant, accurate, came from a reliable source, and was not potentially harmful to users. Moreover, each rater searched for any published studies to establish whether or not there was an evidence base for each app. Furthermore, high interrater agreement was observed between the assessors (intraclass correlation coefficient .904), indicating strong agreement between raters.

## Results

### App Selection

**Figure 1:**
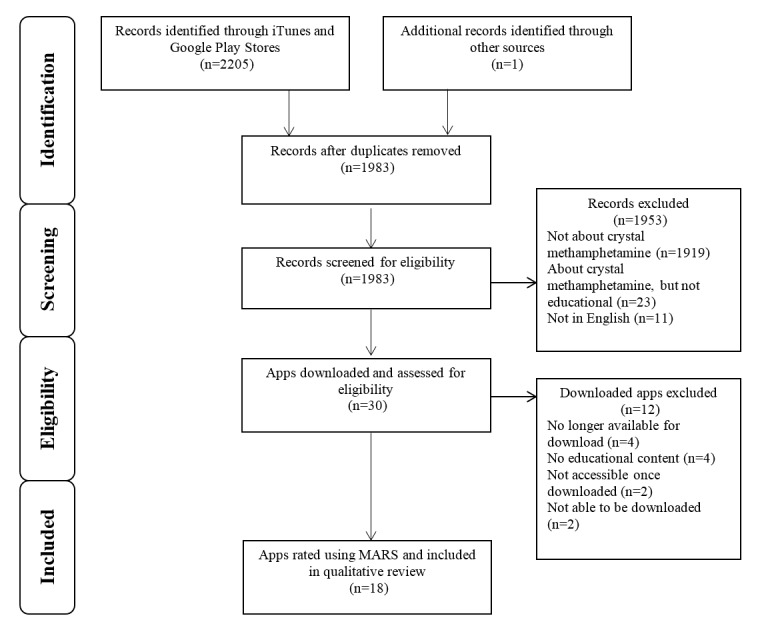
The Preferred Reporting Items for Systematic Reviews and Meta-Analyses (PRISMA) flow diagram of the search strategy and app selection.

Figure 1 displays the Preferred Reporting Items for Systematic Reviews and Meta-Analyses (PRISMA) flow diagram of the full search strategy and the app selection process. A total of 2205 apps were identified through the iTunes and Google Play Stores. Of these, 223 were duplicate apps and 1953 were ineligible (1919 were not about crystal methamphetamine; 23 were about crystal methamphetamine, but not educational; 11 were not in English). A total of 30 potentially relevant apps were downloaded for further screening, and 18 apps were selected for the final data extraction and quality ratings.

### App Classification

Table 1 presents descriptive information about each of the 18 included apps, and Table 2 presents a summary of the app classification across included apps. Just over half of the apps (10/18, 56%), were designed to be used on an iPhone or iPad, with just under half designed for use on the Android platform (8/18, 44%). The majority of apps were freely available for download, and 6 apps (6/18, 33%) required payment before download, with costs ranging from Aus $1.49 to Aus $42.99 per app. The majority of apps were affiliated with a commercial organization (10/18, 56%), 3 of 18 (17%) were affiliated with a university or government department, and 1 app was affiliated with a nongovernment organization. The affiliations of 4 of 18 apps (22%) were unable to be determined because of insufficient information provided by the app developers.

All rated apps included information on methamphetamines (including ice), and the majority (14/18, 78%) also included content about other drugs; only 4 apps exclusively focused on crystal methamphetamine. The most common app feature was the provision of factual information and educational materials (17/18, 94%), followed by offering advice, tips, strategies, or skills training to reduce drug use or related harms (6/18, 33%), assessment of drug use (2/18, 11%), feedback on drug use (2/18, 11%), monitoring or tracking (2/18, 11%), and goal setting (1/18, 5%). None of the identified apps explicitly mentioned a specific theoretical background or utilized evidence-based theory. In terms of technical requirements, 1 app (Street Drugs Organisation) explicitly stated that it needed internet access to function, another reported using automatic sensing (eg, global positioning system), which only functioned in the United States (Drug Sign), and 1 app required a password to log in (ASSIST app). Nearly all apps (17/18, 94%) were targeted to the general population; however, 1 app (Pure Rush) was specifically designed for use by young people aged ≥12 years.

**Table 1:** The classification and content of included apps.

App name	Platform	Cost (Aus $)	Focus: what the app targets	Theoretical background and strategies	Affiliations	Target age groups	Technical aspects
Alcohol + Drugs e-Learning Pro	Android	3.15	Methamphetamines (including ice),other drugs	Information or education	Commercial	General	None listed
ASSIST App	iPhone	Free	Methamphetamines (including ice), other drugs	Assessment; feedback; information or education; monitoring or tracking; advice, tips, strategies, or skills training	Government/university	General	Allows password protection; Requires log-in
Drug Addiction	Android	Free	Methamphetamines (including ice), other drugs	Information or education; Advice, tips, strategies, or skills training	Unknown	General	None listed
Drug Addiction—How to Stop Your Dependence on Drugs	iPhone	1.49	Methamphetamines (including ice), other drugs	Information or education; Advice, tips, strategies, or skills training	Commercial	General	None listed
Drug Addiction: Drugs Handbook	iPhone	Free	Methamphetamines (including ice), other drugs	Information or education	Unknown	General	None listed
Drug Detection App	iPhone	Free	Methamphetamines (including ice), other drugs	Information or education	Commercial	General	None listed
Drug Detection App—Family and Home	iPhone	5.99	Methamphetamines (including ice), other drugs	Information or education	Commercial	General	None listed
Drug Effects Guide & Quiz Game	Android	Free	Methamphetamines (including ice), other drugs	Information or education	Unknown	General	None listed
Drug Sign	iPhone	42.99	Methamphetamines (including ice), other drugs	Assessment; Information or education; Advice, tips, strategies, or skills training	Commercial	General	Uses automatic sensing (eg, global positioning system)
Emergency Responder	Android	2.43	Methamphetamines (including ice)	Information or education; Advice, tips, strategies, or skills training	Commercial	General	None listed
Ice Your Body Belongs to You	iPhone	Free	Methamphetamines (including ice)	Information or education	Commercial	General	None listed
Meth Ice (methamphetamine)	Android	Free	Methamphetamines (including ice)	Information or education	Unknown	General	None listed
Meth Streetdrugs.org	Android	2.54	Methamphetamines (including ice)	Information or education	Commercial	General	None listed
National Drugs Campaign	iPhone	Free	Methamphetamines (including ice), other drugs	Information or education	Government	General	None listed
Overdose Aware	iPhone	Free	Methamphetamines (including ice), other drugs	Information or education	Nongovernment organization	General	None listed
Pure Rush	Android	Free	Methamphetamines (including ice), other drugs	Information or education	Government/university	12 years+	None listed
Street Drugs Organisation	Android	Free	Methamphetamines (including ice), other drugs	Information or education	Commercial	General	Needs Web access to function
Triggr Health—Support for Reducing Drinking/Using	iPhone	Free	Methamphetamines (including ice), other drugs	Feedback; Monitoring or tracking; goal setting; advice, tips, strategies, or skills training	Commercial	General	None listed

**Table 2:** The summary of features of included apps.

Feature		Apps (N=18), n (%)
**Platform**		
	Android	8 (44)
	iPhone	10 (56)
**Cost (Aus $)**		
	Free	12 (67)
	<6	5 (28)
	>40	1 (6)
**Focus**		
	Methamphetamines (including ice)	4 (22)
	Methamphetamines (including ice) and other drugs	14 (78)
**Theoretical background and strategies**		
	Information or education	17 (94)
	Advice, tips, strategies, or skills training	6 (33)
	Assessment of drug use	2 (11)
	Feedback on drug use	2 (11)
	Monitoring or tracking	2 (11)
	Goal setting	1 (6)
**Affiliations**		
	Commercial	10 (56)
	University or government	3 (17)
	Nongovernment organization	1 (6)
	Unknown	4 (22)
**Target age groups**		
	General population	17 (94)
	>12 y	1 (6)
**Technical aspects**		
	None	15 (83)
	Password protection or requires log-in	1 (6)
	Automatic sensing (eg, global positioning system)	1 (6)
	Web access required	1 (6)

### App Quality Ratings

**Figure 2:**
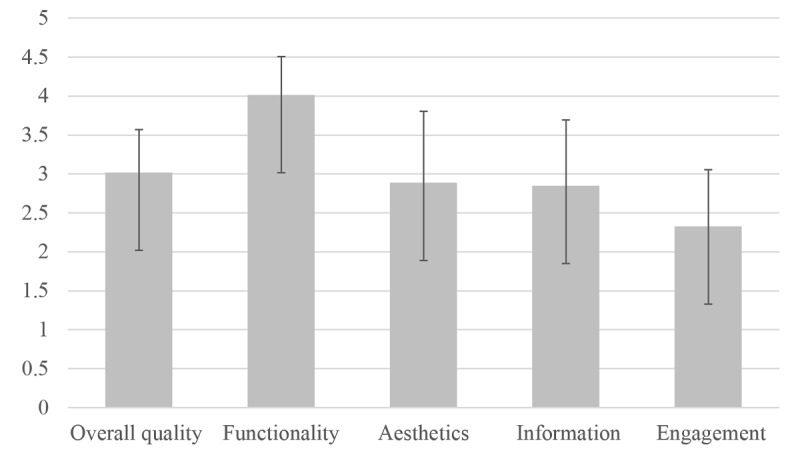
The means and SDs for the overall quality rating and Mobile Application Rating Scale subscales for the included apps.

Table 3 presents the mean subscale scores and the overall mean quality ratings on the MARS for each included app. Figure 2 presents the overall mean scores on the MARS subscales for all included apps. The mean MARS quality total score for all rated apps was 3.0 (SD 0.6), indicating *poor* to *acceptable* quality. A cutoff of 3.0 has been established as a minimum acceptability score [34], and nearly half (8/18, 44%) of the rated apps failed to meet this threshold. Only 2 apps (*Pure Rush* and *Triggr Health)* achieved an overall quality score >4, indicating they were of *good* quality. Similarly, the mean subjective quality total was *poor* to *inadequate* at 1.8 (SD 0.8). No apps received a rating of ≥4 on both the overall quality and the subjective quality, indicating there were no apps of overall *good* or *excellent* quality when both scales were considered together.

When examining the mean MARS subscale scores for included apps, we observed considerable variability in mean ratings of engagement, functionality, esthetics, and information quality. The overall mean scores were the highest for functionality (mean 4.0, SD 0.5), with 44% of apps (8/18) achieving a score of ≥4, indicating *good* functionality and reflecting high scores in particular on the subscale item ease of use, where only 1 app failed to achieve a rating of ≥4. Mean scores were the lowest for engagement (mean 2.3, SD 0.7), with only 1 app scoring ≥3 and none scoring ≥4. Scores were particularly low for interactivity and customization with the majority of apps rated as 1 (*inadequate*) on one (6/18) or both (9/18) of these items.

**Table 3:** Mobile Application Rating Scale ratings.

App name	Engagement	Functionality	Esthetics	Information	Overall quality total	Subjective quality total
Alcohol + Drugs e-Learning Pro	3.5	4.1	3.5	3.6	3.7	2.1
ASSIST App	2.3	3.5	2.3	4.0	3.0	2.1
Drug Addiction	1.6	4.1	2.0	1.8	2.4	1.0
Drug Addiction—How to Stop Your Dependence on Drugs	2.3	4.4	2.2	1.8	2.7	1.3
Drug Addiction: Drugs Handbook	1.3	4.3	1.3	2.0	2.2	1.0
Drug Detection App	2.0	3.4	3.0	3.2	2.9	1.9
Drug Detection App—Family and Home	2.0	3.9	3.0	3.2	3.0	2.1
Drug Effects Guide & Quiz Game	2.6	2.9	2.8	1.4	2.4	1.0
Drug Sign	2.1	3.5	3.7	2.7	3.0	1.9
Emergency Responder	2.0	4.0	2.7	3.2	3.0	1.8
Ice Your Body Belongs to You	2.9	4.0	3.0	2.8	3.2	1.5
Meth Ice (methamphetamine)	1.8	4.3	2.3	2.2	2.6	1.3
Meth Streetdrugs.org	1.8	4.4	2.3	2.1	2.6	1.5
National Drugs Campaign	1.8	3.5	1.8	4.1	2.8	2.1
Overdose Aware	2.2	4.5	3.5	4.0	3.6	2.9
Pure Rush	3.6	4.6	5.0	3.8	4.2	3
Street Drugs Organisation	2.1	4.3	3.0	2.7	3.0	1.0
Triggr Health—Support for Reducing Drinking/Using	4.0	4.8	4.5	2.9	4.0	3.6

**Figure 3:**
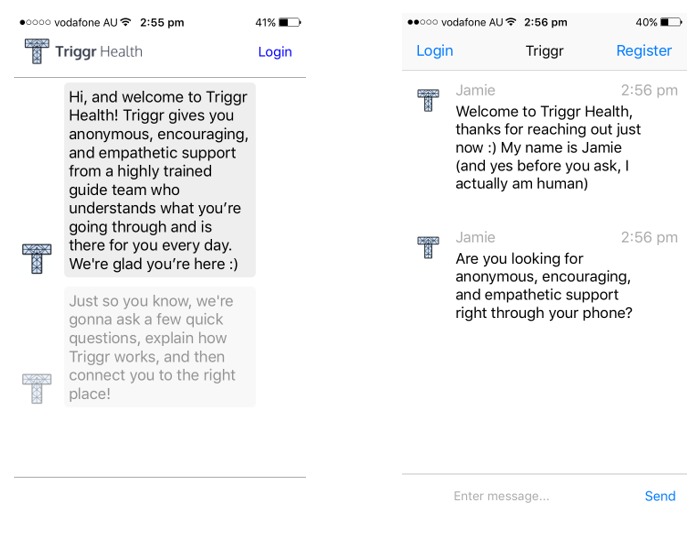
Screenshots of the Triggr Health App. (Source: Triggr Health - Support for Reducing Drinking/Using iPhone App Version 2.19.4. Developer: Triggr LLC. Licensed under fair use).

In addition, mean scores for esthetics were poor with only 2 apps scoring ≥4, indicating a rating of *good*. The layout was rated as, at least, acceptable for 67% of apps (12/18); however, graphics and visual appeal were rated as *poor* to *inadequate* in 6 and 9 out of the 18 included apps, respectively. While the overall mean score for the information quality was low for included apps (mean 2.8, SD 0.8), there was considerable variability across apps, with 2 apps scoring <2 and 3 apps scoring ≥4. Apps that achieved lower scores on the information quality subscale received low scores across subscale items, including inaccurate descriptions in the app store, poorly defined goals, poor-quality content, poor quantity of information provided (too much or too little), the app was unlikely to come from a credible source (ie, was most likely developed by a source with a vested interest, for example, commercial business), visual information was incorrect or confusing, and there was no available evidence base for these apps in the form of published evaluations.

### Features of the Top-Ranked Apps

Although no apps were identified as having *excellent* overall quality, 2 apps achieved a *good* overall quality rating, with *acceptable* subjective quality ratings (Triggr Health—Support for Reducing Drinking/Using and Pure Rush). Triggr Health is a commercial app developed by a US company Triggr Health (Figure 3). The app targets addiction recovery and is focused on reducing substance dependence for a range of drugs, including crystal methamphetamine. The app connects users to a real-time behavioral change “guide” who interacts with users to set personalized goals and recovery plans. In addition, it utilizes predictive machine learning algorithms to identify smartphone use patterns and “check in” with users about their goals and recent activity. To date, no efficacy or effectiveness data exist to indicate that the Triggr Health app can prevent or reduce crystal methamphetamine use and harms.

**Figure 4:**
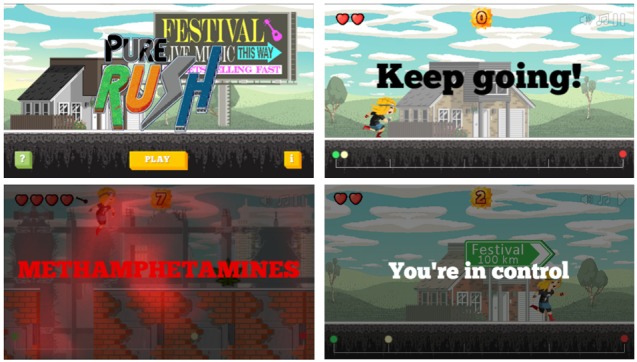
Screenshots of the Pure Rush app. (Source: Pure Rush Android App Version 2. Developers: National Health and Medical Research Council Centre of Research Excellence in Mental Health and Substance Use and 2and2. Licensed under fair use).

Pure Rush is a serious educational game [36] for young people including information about cannabis, hallucinogens, crystal methamphetamine, and 3,4-methylenedioxy-methamphetamine (MDMA or ecstasy; Figure 4). In the app, users select a character and navigate through a music festival, with the goal of the game to avoid “running into” drugs. The app provides educational information about the negative effects of different drugs in an engaging manner. Pure Rush has been evaluated in one published peer-reviewed study [37], in which 281 young people (aged 13-16 years) were randomly allocated to receive a lesson involving Pure Rush or to an active control lesson; this evaluation found that the app was enjoyable to use, with both conditions associated with a marked increase in drug knowledge from pre- to posttest. There was some evidence that females who received Pure Rush showed greater knowledge gains compared with those in the control.

While this evaluation is promising, it focused on overall illicit drug knowledge and its effects on drug usage and, specifically, crystal methamphetamine usage, is not known. Furthermore, it should be noted that the app was designed to be implemented alongside a companion booklet that provides additional information (normative education, assertiveness skills, and corrections of common misperceptions), rather than as a standalone educational tool [37].

Other than for functionality, very few apps achieved ratings of *good* on any of the MARS subscales (information quality, engagement, and esthetics). Three apps achieved ratings of *good* on the information quality subscale—the ASSIST app, National Drugs Campaign app, and Overdose Aware app. Both the ASSIST and National Drugs Campaign apps are affiliated with a university or government department, and the Overdose Aware app is affiliated with a nongovernment organization. The ASSIST app was developed by the University of Adelaide and provides information, self-assessment, and links to support services for a range of drugs including amphetamines. Information covers the physical and mental effects of different drugs, harm reduction information, and links to relevant support services. The information provided is of high quality, but focused on a wide range of drugs and amphetamines as a broad class of drugs. The National Drugs Campaign app is a companion app to the Australian Government National Drugs Campaign website. It provides information about a range of drugs, including crystal methamphetamine, and links to external support sites. Overdose Aware was developed by the Pennington Institute and targets overdose education, including information about what an overdose is and recognizing overdose symptoms. It focuses on 4 classes of drugs—stimulants, depressants, opioids, and alcohol. None of these apps achieved a score of *acceptable* or above for engagement.

## Discussion

### Principal Findings

This study aimed to systematically review the existing apps to determine the existence, composition, and quality of educational smartphone apps about methamphetamines, including ice. We used the MARS to identify descriptive features and content of the apps and systematically rate quality in terms of engagement, functionality, esthetics, and quality of information. We examined app store descriptions for 1983 apps, downloaded and assessed 30 apps for eligibility, and rated 18 for content and quality. Only 4 identified apps focused exclusively on crystal methamphetamine, with the majority focusing on illicit drugs in general. The majority of apps reviewed focused on providing information or education, one-third provided advice, tips, strategies, or skills training, with few offering other features such as self-assessment of drug use, feedback, and monitoring or tracking of drug use. Functionality was rated as *acceptable* to *good* on the majority of apps; however, the overall quality was low, with only 2 apps achieving a rating of *good* on overall objective quality and *acceptable* on subjective quality according to the MARS. Ratings of the information quality varied considerably with only 3 apps achieving a rating of *good* in this domain, despite 94% of apps aiming to provide accurate information and education.

### Comparison With Prior Work

Several findings warrant further comment. First, the mean overall MARS quality rating of 3.0 is commensurate with other apps of this scale in the health field [35,38,39]. Similarly, the finding that few apps consistently scored highly across all MARS dimensions also mirrors findings of previous studies that have assessed the quality of apps using this scale [35,38-40]. Bardus et al [38] used the MARS to assess the quality of commercial apps to assist with weight management and found that the information quality was the lowest scoring subscale for included apps and noted the lack of evidence-based content as a key driver of poor quality. In this study, the information quality was the second lowest scoring dimension after the engagement dimension. Notably, of apps that achieved either an overall quality rating of *good* (n=2) or a rating of *good* in terms of the information quality (n=3), only one carried a commercial affiliation (Triggr Health). The remainder were affiliated with either a university or government department (Pure Rush, ASSIST app, and National Drugs Campaign), or a nongovernment organization (Overdose Aware). Previous reviews have noted that the involvement of health professionals or other experts in health-related app development is often lacking or difficult to assess [29,33]. It is likely that this has contributed to low quality, particularly in terms of the evidence base of the apps and points to the need to involve experts in the development of good-quality apps in this area. In addition, it highlights the need to clearly identify affiliations and the evidence base used in the development of apps so that consumers can make informed choices [33]. Reviewers of apps in the substance use field have suggested clearer guidelines for the public [25], more stringent regulations [32], or more visible means of identifying quality apps [33] to improve consumer choice. In the process of conducting this review, >1900 irrelevant apps were identified in the searches; this has significant implications for people who are genuinely seeking help or information about crystal methamphetamine or other drugs and underscores the need for both better-quality apps on the market and better ways of guiding consumer choice about these apps.

Even within the context of crystal methamphetamine apps that do provide high-quality and evidence-based information, the lack of interactivity and engaging features represents a significant lost opportunity for reach and impact [31]. Only 3 apps offered interactive features such as monitoring, tracking, or goal setting. A previous review of smartphone apps to manage alcohol use, found that features such as tracking and tailoring were markedly associated with the app popularity (in terms of downloads) and user-rated quality [30], providing support for the inclusion of these features in the future development of apps. Similarly, a previous review of apps to support app users’ weight management using the MARS also found that the overall app quality correlated with the number of different techniques or interactive features available [38]. One of the challenges in developing engaging, evidence-based apps is that despite the fact that government or university developed apps may be more likely to be based on evidence and involve health professional and other expert input, they are often competing with higher budget apps in the commercial space [30]. In this review, Triggr Health was the only app to achieve a score of *good* in terms of engagement, and this was also the only commercial app to achieve an overall quality rating above *acceptable*. It did, however, achieve its lowest rating in the area of information quality, further highlighting the need to balance credibility and accuracy of information with highly specialized technical features and interactivity.

The final point worthy of discussion is a large number of entertainment-based apps about crystal methamphetamine that were identified in the initial searches of the app stores. For example, several apps capitalized on the popularity of the television show, *Breaking Bad,* in which the central character manufactures and deals crystal methamphetamine. Although some of the available apps were seemingly harmless, for example, trivia about the episodes, downloadable artworks, and ringtones, others included game functionality where users could virtually cook methamphetamine or search for methamphetamine crystals. These latter apps highlight the influence of the media and popular culture on commercial app development as a potential public health concern, especially for young people or vulnerable groups. Gaming technology does hold great potential for the app development industry, especially for apps related to addiction and other health issues [31,37]; however, it is critical that app developers achieve the right balance among gamification, evidence, and quality of information.

### Limitations

There are several limitations to this review that warrant discussion. First, the app market is highly dynamic. The availability of apps changes regularly, and this review can only offer a snapshot at one point in time. Within this context, it should also be noted that app stores do allow publishers to restrict distributions to particular countries. A previous systematic review of suicide apps available in Australia found 100% concordance between available apps across iOS app stores in a number of countries [23]. Nonetheless, this review focused only on apps available for download in Australia. Second, although this review focused on systematically rating the content and quality of apps, it does not provide information about the effectiveness of the apps in promoting accurate messages about crystal methamphetamine or preventing or reducing use and harms. One of the apps reviewed had been subject to a randomized controlled trial [37]; however, rigorous evaluations of app effectiveness in the illicit substance use field are rare [12,13]. While scientific evaluation of the effectiveness of apps is important, the speed with which the app market and the technology on which it is based changes rapidly and the length of time needed to conduct and publish randomized controlled trials presents a considerable challenge to researchers [12]. Some reviewers have suggested it might be useful for the mobile health field more broadly to focus on more pragmatic and less traditional modes of evaluation to assess the effectiveness of apps and other mobile health interventions to enable the field to build the evidence base more quickly [20,25].

Third, a relatively small number of apps met the inclusion criteria for the review, limiting the generalizability of the findings and indicating a need for further research in this area. For example, although the review points to general principles to guide the future development of evidence-based, high-quality apps, this study is not able to inform the specific circumstances under which these features might best apply. Along similar lines, very few of the included apps exclusively targeted crystal methamphetamine; this reflects the current app landscape and demonstrates a gap in the app market for apps specifically targeting crystal methamphetamine. The large number of apps targeting substances such as cannabis, on the other hand, points to a need for future systematic reviews of apps that target substances other than methamphetamine to inform a changing and fast-growing area. Finally, the minimum time period of a single 10-minute session per app may not be sufficiently long enough to comprehensively evaluate app features, such as tracking use over time and behavioral change, which were present in 2 of the included apps.

### Conclusions

Crystal methamphetamine is a high-impact drug that is associated with considerable harms and high levels of community concern. Importantly, the majority of people using crystal methamphetamine do not want to engage with traditional treatment or support services for fear of stigma and concerns about relevance [41]. This makes the increasing availability of app-based information and support for crystal methamphetamine (and other substances) critically important. This study was the first to systematically review the quality of available apps focusing on methamphetamine, including “ice.” Despite the fact that many available apps purport to be about crystal methamphetamine, most do not offer educational content. Of those that do, most have not been subject to rigorous evaluations, they vary in quality, and despite having good functionality, few are likely to engage the public. Given the enormous potential of smartphone apps to promote positive and accurate public health messages and to prevent use and harms, this represents a significant opportunity for future development.

## Abbreviations

MARS: Mobile Application Rating Scale
